# What Is the Truth about Bradford?

**Published:** 1921-01-29

**Authors:** 


### WHAT IS THE TRUTH ABOUT BRADFORD?

From the time when the Ministry of Health agreed to the handing over of St. Luke's Poor-law Hospital to the Health Committee of the Bradford Corporation, Dr. Addison's critics have used and misused what might have been a technical breach of the law to harass the Minister of Health. The Minister was from the first quite willing to admit the possible irregularity of the step, but secured many sympathisers by stating that he was influenced by the shortage of hospital beds in Bradford. Since then the opponents of municipal general hospitals must have been a little perplexed by the Minister's silence under a smart storm of accusations of lawlessness.

We do not at the moment propose to say much of the principle of the step at Bradford, except that, for the sake of the ratepayer, we hope it will not be too readily copied elsewhere. But we do heartily wish that somebody who is not an obvious partisan would tell us the truth about Bradford. What is St. Luke's Hospital really like at the present moment? Is it, as described by Mr. J. Basil Hall, of the honorary staff of the General Infirmary, "the same old obsolete group of buildings which the Local Government Board condemned as unfit twenty years ago," which, after requirements for public-health purposes have been satisfied, leaves nothing for general surgery and medicine? Or is it, as described by Dr. B. Holroyd Slater, the Medical Superintendent, a building no portion of which has been condemned by the Local Government Board, with three modern blocks, completed sixteen years ago, having accommodation for 156 adults, 80 children, and 45 maternity cases, and with a first-class operating-theatre; with three other pavilions built within the last four years, each accommodating 100 patients?

Then, again, what are the hospital needs of Bradford? Mr. Basil Hall appears to consider that the 361 voluntary beds suffice. Dr. Peter Macdonald, of York, a municipal hospital advocate, considers that with 514 at St. Luke's added there will be no surplus above the needs of a population which cannot be less than 300,000. Possibly each is right—and wrong. Mr. Basil Hall must know that in the future, if we are not too bankrupt to afford it, the hospital will not wait for the patient to become desperately ill before he is admitted, and that the general health of the public will greatly benefit by the treatment of early conditions of disease, thereby requiring many more beds.

We eagerly desire the non-partisan statement of the whole Bradford position, because it will teach us much about the hospital position in other manufacturing cities, where difficulties are being solved in other ways. The voluntary hospital surgeon asks for "a first-class general infirmary and a municipal hospital to work with it." The Poor-law Medical Superintendent fears that "co-operation in this case implies the conversion of St. Luke's, with its splendid wards and equipment, into a dump on which the Infirmary could shoot its patients after giving them the more exciting portions of their surgical treatment." To borrow a homely and useful saying from our American friends, is it not possible for these two gentlemen and the systems they represent "to get together"?

